# Exploring right ventricular function applicability in a prediction model to identify preterm infants with early bronchopulmonary dysplasia (REPORT-BPD study): a mixed-methods observational cohort feasibility study protocol

**DOI:** 10.1186/s40814-022-01201-1

**Published:** 2022-12-09

**Authors:** Wisam S. Muhsen, Eirik Nestaas, Joanne Hosking, Jos Latour

**Affiliations:** 1 Neonatal Intensive Care Unit, University Hospital Plymouth NHS Trust, Plymouth, UK; 2 Faculty of Health, University of Plymouth, Plymouth, UK; 3 Institute of Clinical Medicine, University of Oslo, Oslo, Norway; 4 Clinic of Pediatrics and Adolesence, Akershus University Hospital, Nordbyhagen, Norway; 5 Medical Statistics, Faculty of Health, University of Plymouth, Plymouth, UK

**Keywords:** Right ventricular function, Bronchopulmonary Dysplasia, Prematurity, Neonates, Echocardiography

## Abstract

**Background:**

Bronchopulmonary dysplasia (BPD) is a chronic disease that affects the immature lungs of preterm infants. Infants born before 32 weeks of gestation are at a greater risk of developing BPD due to the need for respiratory support with higher oxygen requirement.

Pulmonary vascular remodelling in early BPD can impose an additional burden on the right ventricle (RV) and RV dysfunction.

This protocol outlines the study design and aims to formulate a prediction model to identify early BPD through the data generated from echo scans analysis.

**Methods:**

The mixed-methods observational cohort feasibility study, which comprises three work-packages (WPs), will be conducted at the regional neonatal unit, University Hospital Plymouth, Plymouth, UK. WP-I will recruit 40 preterm infants; each participant will have two heart scans performed in the first ten days after birth (DABs).

WP-II will collect the documentation of the participating preterm infants’ parents in the study neonatal unit diaries in the first 10 DABs. WP-III will involve semi-structured interviews of 10–15 parents of participating preterm infants and 10–15 health professionals who participated in WP-I.

The study recruitment will be conducted over 18-months. The start date is 01 June 2022. WP-I and WP-II recruitment will occur during this period, while WP-III recruitment will occur during the second half. The results are expected to be submitted for publication by mid-2024.

**Discussion:**

This paper outlines the study design. If the study successfully identifies the most sensitive echo parameter in recognising the RV dysfunction associated with early BPD, it will be an important finding in constructing an early BPD prediction model.

**Trial registration:**

ClinicalTrials.gov Identifier is NCT05235399

## Background

Bronchopulmonary dysplasia (BPD), also known as chronic lung disease, was first identified in 1967 [1]. This condition mainly affects the immature lungs of preterm infants. In the UK, about one third of preterm infants born before 32 weeks of gestation are diagnosed with BPD [2]. Hence, the National Neonatal Audit Programme (NNAP) recommendations urge healthcare professionals to identify potential changes in practices that can improve the respiratory function of preterm infants [2].

BPD negatively affects the normal growth and development of the immature lungs’ alveoli and vascular bed [3]. A recent study, despite its limitation of being an animal study, showed a crucial finding when it demonstrated that changes in the pulmonary capillary bed occur prior to those in the lungs’ parenchyma, which supports the vascular theory; the pathological process of BPD starts in the pulmonary vascular bed [4]. The pulmonary vascular remodelling in BPD affects preterm infants, resulting in a rise in the vascular tone, altered reactivity, vasoconstriction and increased pulmonary vascular resistance [5]. These changes generate a higher right ventricle (RV) afterload pressure. Subsequently, the chronic increase in RV afterload pressure and hypoxic episodes can result in RV dysfunction, hypertrophy and, in severe cases, RV failure [6]. Furthermore, severe forms of BPD are associated with a higher incidence of pulmonary arterial hypertension [7], which is associated with significant co-morbidities and high mortality rates [8].

Additionally, BPD commonly causes adverse effects, such as poor health, longer hospital stays, prolonged need for oxygen therapy, neurodevelopmental delay and a higher rate of death in affected preterm infants [9, 10].

The diagnostic definition of BPD is mainly based on the oxygen requirements and respiratory support at 28 days and 36 weeks of postmenstrual age (PMA) [11–13] without any inclusion of pathophysiological changes of early BPD [14].

The recent classifications of BPD divide the disease into grades I, II, III and/or III(A) according to its severity [12, 13]. These classifications [12, 13] include additional modalities of respiratory support, such as Humidified Heated High Flow Nasal Cannula, which was not included in the previous definition by Jobe and Bancalari [11]. Irrespective of the diagnostic definition used, the formal diagnosis and severity of BPD will not be established until the preterm infant is 36 weeks of PMA or at discharge (if the infant is discharged prior to that).

Czernik et al. [15] demonstrated a persistently elevated myocardial performance index of the RV in the first 2 weeks after birth (WABs) in preterm infants with incipient BPD. The identification of RV dysfunction associated with early BPD through echocardiography (echo) scan examination in these 2 weeks can serve as a marker for detecting preterm infants with the condition [15, 16].

The data obtained from 1735 infants born between 23 and 30 weeks of gestational age showed that the proportion of these infants needing oxygen decreases from birth to the 7th day after birth (DAB). This is followed by a steep rise in the number of infants requiring oxygen in the same cohort at the start of the second WAB [17]. These studies indicated that pathological pulmonary changes start as early as the first WAB [15, 17].

This paper describes the protocol of the proposed clinical study, which examines the applicability of RV functional assessment in an early BPD prediction model. This study protocol details three work packages (WPs): WP-I, which comprises the performance of two echo scans at the 5th and 9th DABs; WP-II, which is an embedded qualitative research project that aims to examine the parental perception of their participating preterm infants’ health; this is performed by analysing the parental written notes in the neonatal unit diary in the first 10 DABs; and WP-III, which is a further qualitative research project that involves conducting semi-structured interviews with 10–15 parents of the participating preterm infants and 10–15 health professionals who are involved in the initial phases of this study.

This study (REPORT-BPD) aims to capture the burden exerted on the RV by early BPD and its associated pulmonary vascular changes by performing functional echo examinations at two time points—5th and 9th DABs—and identifying the most sensitive echo scan parameter to diagnose RV dysfunction. The findings will be used to construct an early BPD prediction model.

The embedded qualitative research components are added to this project as qualitative research methodologies are commonly used by researchers to explore the shortfalls and adjustments required for the planned larger trial [18].

1. Feasibility outcomes and progression criteria
  1.1. Primary outcome:
    1.1.1. The feasibility of constructing a prediction model of BPD through diagnosing RV dysfunction associated with early BPD
  1.2. Secondary outcomes:
    1.2.1. Identification of the most sensitive echo parameter(s) to diagnose early BPD-associated RV dysfunction
    1.2.2. Establishing if adjustments are needed to the study procedures, such as screening, recruitment and performance of echo scans, to maximise recruitment and minimise data loss
    1.2.3. Establishment of recruitment and retention rates for preterm infants with and without BPD, i.e. cases and controls
    1.2.4. Obtaining descriptive statistical data about the study cohort and the selected echo parameter(s) will facilitate the estimation of the sample size needed for the definitive large study
  1.3. Progression criteria:
  The following progression criteria should be met
  1.3.1. Constructing a BPD prediction model based on a sensitive echo parameter in detecting early BPD-associated RV dysfunction is a key criterion to progress to a definitive large study
  1.3.2. Satisfactory recruitment and retention rate of preterm infants with and without BPD
  1.3.3. Logistical challenges in performing the echo scans; two should have been performed on most of the recruited preterm infants (≥ 80)
  1.3.4. Most of the echo scans (≥ 80%), especially the Tissue Doppler Imaging (TDI) and the speckle tracking clips and images, should be suitable for analysis by the cardiology analysis system (TomTec, Germany)

## Methods

**Fig. 1:**
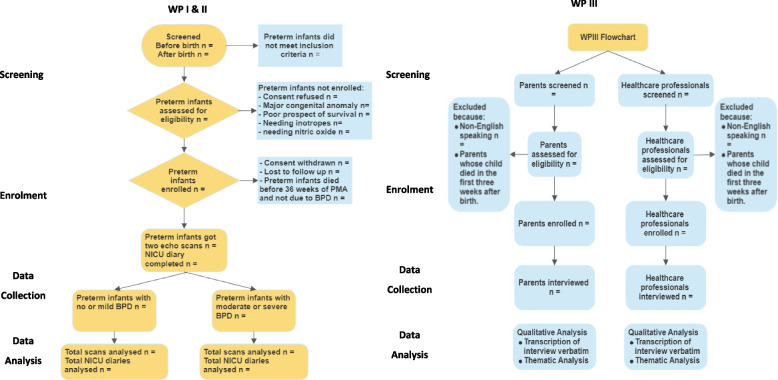
Study flow charts for all work packages

The study procedures and flow for all three WPs, from screening to data analysis, are summarised in Fig. 1 and Table 1.

**Table 1:** Study procedures for all work packages

**WP-I and WP-II**									
**Procedures**	**Antenatally**	**1st DoPL©**	**2nd DoPL©**	**Data collection**					
				**T0**	**T1**	**T2**	**T3**	**T4**	**T5**
Screening and eligibility assessment	**√**^a^	**√**	-	-	-	-	-	-	-
Initial conversation with the research team	**√**^a^	-	**√**	-	-	-	-	-	-
Informed consent	-	-	**√**	-	-	-	-	-	-
Demographics (when consent obtained)	-	-	**√**	-	-	-	-	-	-
Echo scan on 5th DoPL	-	-	-	**√**	-	-	-	-	-
Echo scan on 9th DoPL	-	-	-		**√**	-	-	-	-
Working weight	-	-	-	**√**	**√**	-	-	-	-
Vital signs including systolic, diastolic, and mean Blood Pressure (BP)	-	-	-	**√**	**√**	-	-	-	-
Collection of NICU parents/patients’ diary copies	-	-	-	-	-	**√**	-	-	-
Collection of data related to outcomes/diagnosis of BPD	-	-	-	-	-	-	-	**√**	-
**WP-III**									
**Procedures**	**Antenatally**	**1st DoPL©**	**2nd DoPL©**	**Screening, consenting and data collection**					
				**T0**	**T1**	**T2**	**T3**	**T4**	**T5**
Screening and eligibility assessment	-	-	-	-	**√**	-	-	-	-
Initial conversation with the research team	-	-	-	-	**√**	-	-	-	-
Informed consent	-	-	-	-	**√**	-	-	-	-
Parents semi-structured interviews	-	-	-	-	-	-	**√**	-	-
Clinicians semi-structured interviews	-	-	-	-	-	-	-	-	**√**

© Day(s) of postnatal life (DoPL)

T0 = 5th DoPL

T1 = 9th DoPL

T2 = 10th DoPL

T3 = 10th–17th DoPL

T4 = 36 weeks of PMA or gestation in weeks if discharged home earlier than 36 weeks PMA

T5 = semi-structured interviews will be conducted within one month after obtaining consent

^a^If possible

### WP-1

#### Objectives

- To assess the feasibility and acceptability of the study procedures (e.g. recruitment, echo scan performance, data collection, storage and analysis)
- To identify the sensitive echo parameters in assessing the RV function of the heart to be included in a prediction model to identify early BPD in premature infants

#### Study design and settings

This is a longitudinal, observational, cohort and feasibility study that will be conducted at the regional tertiary neonatal intensive care unit (NICU) at University Hospitals Plymouth NHS Trust (UHP), Plymouth.

#### Recruitment and sample size

The exploratory nature of this feasibility study sets a realistic target of recruiting 40 preterm infants who are born before 32 weeks of gestation.

The regional NICU at the UHP admitted 89, 101 and 69 preterm infants who were born before 32 weeks PMA in the years 2018, 2019 and 2020, respectively. Based on the careful examination of the admission data of 2018, two thirds of these preterm infants met the inclusion criteria of the proposed study, with one third of them developing moderate-to-severe BPD.

Therefore, the proposed feasibility study’s realistic sample size is 40 preterm infants over an 18-month recruitment period. Following are the participants whose data will be subjected to the trial analysis:

1. Infants who survived until the gestational age when the diagnosis of no BPD or severe BPD can be established
2. Infants who died before the set gestational age (36 weeks PMA) when severe BPD was identified as the main cause of death

The screening for potential recruits against the inclusion and exclusion criteria (Table 2) will be conducted at the following two timepoints:

- Timepoint 1: If possible, antenatal screening will be conducted by the clinical neonatal team by reviewing the maternal medical notes. Then, the neonatal research team will be informed so that the initial conversation can occur antenatally if the unborn infant is deemed a potential recruit.
- Timepoint 2: If screening is not possible antenatally, then parents of the suitable preterm infants will be approached in the second DAB of their children.

**Table 2:** Inclusion and exclusion criteria for WP-I

Inclusion criteria	Exclusion criteria
1. Born at < 32 weeks gestational age. 2. The echo scans are indicated as per the recommended neonatal practice [19]: i. Preterm infants who require mechanical ventilation or non-invasive respiratory support (CPAP ≥ 4 cm H_2_O, high flow ≥ 4 L/min) ii. Detection of a cardiac murmur in the first 3 days after birth iii. Assessment of patent ductus arteriosus (PDA) iv. Preterm infants have central line in-situ, so echo is needed to assess line position	1. Preterm infants with major congenital anomalies, such as pulmonary hypoplasia or congenital heart disease (except PFO or PDA) 2. Preterm infants with a poor prospect of survival 3. Preterm infants whose parents do not consent 4. Preterm infants who are still in need of blood pressure supporting medications, such as, (inotropes) when the study echo scans are due 5. Preterm infants need inhaled nitric oxide gas when the study echo scans are due

*PDA* patent ductus arteriosus, *PFO* patent foramen ovale

All parents will be spoken to in person by the neonatal research team, who are also part of the neonatal clinical team in the antenatal wards in the delivery suite antenatally or in the neonatal unit postnatally. A clear verbal explanation will be provided about the trial and why the parents and their preterm infants are invited to participate. They will also be given a participant information sheet (PIS) and an informed consent form (ICF). If the parents wish for their preterm infant to be recruited for the study when feel well-informed and satisfied with the explanations provided, they will be asked to sign the ICF.

Consent will be sought for WP-I and WP-II from the parents of the eligible-for-inclusion preterm infants on the second DAB. All participants will be informed that they are free to withdraw from the study at any time without giving reasons and without prejudicing further treatment.

A delegation log will be maintained to ensure that the medical practitioners registered as study delegates participating in the consenting process of this study are duly authorised, have completed the Good Clinical Practice (GCP) training, are experienced clinicians and competent to participate according to the ethically approved protocol, principles of GCP and Declaration of Helsinki [20].

#### Data collection and analysis

Echo scans and clinical and demographic data will be collected.

Demographic data, including birth gestation in weeks, birth weight, sex, Apgars, chorioamnionitis, maternal hypertension, antenatal steroids, and magnesium sulphate, will be collected.

Functional neonatal echo scans will be performed, collected and stored on the 5th and 9th DABs of the recruited preterm infants. They will be collected as per the developed echo image acquisition guide, which is in compliance with the expert consensus statement produced by the American Society of Echocardiography in collaboration with the European Association of Echocardiography and the Association for European Pediatric Cardiologists [21]. Numerous echo parameters, such as global and segmental strain and strain rate for right and left ventricles (RV and LV), tissue Doppler imaging (TDI) of RV free wall, septal and LV lateral wall to measure the S, E’ and A’ waves together with isovolumic contraction and relaxation times, will be collected. Other echo parameters related to pulmonary arterial pressure, cardiac function and ductus arteriosus (DA) assessments such as tricuspid regurgitant jet, pulmonic valve insufficiency, pulmonary artery acceleration time (PAAT), right ventricle ejection time, tricuspid annular plane systolic excursion (TAPSE), mitral annular plane systolic excursion (MAPSE), shortening fraction of the LV and DA size and shunt assessment, will be collected too. Also, right ventricular systolic pressure and myocardial performance indices of RV and LV, will be calculated.

These scans will be analysed using a specialised cardiology analytic system (TomTec, Germany).

In addition, clinical data, vital signs, current weight and ventilatory support will be collected.

The distribution of all continuous variables will be visualised to check for normality and summarised using appropriate descriptive statistics. Preterm infants will be categorised into two groups: (i) moderate or severe BPD and (ii) mild or no BPD.

Velocities and displacements are dependent on cardiac size. Therefore, it is crucial to normalise the measurements taken for cardiac size [22]. The left ventricle septal length will be used to normalise all echo measurements of interest.

The collected echo parameters measurements, clinical data, vital signs, current weight and ventilatory support will be analysed and compared between the cases and controls. Also, if any, missing data will be quantified so appropriate modifications will be implemented to improve the data collection process in a definitive large study.

The comparison between the two groups will be further assessed using the t-tests conducted for normally distributed continuous variables and Mann–Whitney *U* tests for non-normally distributed variables. Chi-squared tests will be conducted to compare categorical variables between groups. The generated descriptive statistical data will inform the sample size estimation for the definitive large study. Also, establishing the most sensitive echo parameters in recognising RV dysfunction will facilitate the construction of the BPD prediction model.

The prediction model will mainly be consisted of the most sensitive echo parameter(s) with incorporation of the gestational age in weeks at birth, oxygen requirements and ventilatory support at the time of the echo scans.

### WP-II

This WP will examine the parental notes written in their preterm infants’ NICU diary. The parental diary in the intensive care setting is used to help parents cope with the situation by writing down their feelings [23, 24]. There is a paucity of research project regarding the use of parental perceptions recorded in the NICU diary about their children’s health. Therefore, the diary recordings of the participating preterm infants’ parents about the respiratory function changes observed by the parents in the first 10 DABs will help in understanding the significance of parental perception of the progression of their infants regarding the respiratory function. Mining and examining these data will highlight the strength of the correlation between the parental observations of their children’s health in the first 10 DABs in recognising early BPD symptoms.

#### Objectives

To understand parental perceptions regarding their preterm infants’ health progression in the first 10 days of postnatal life and its correlation with the echo scans findings.

#### Study design and settings

This is an embedded qualitative study that explores the parental perception of their infants’ health written in a diary.

This study will be conducted in the regional tertiary NICU, UHP, Plymouth.

#### Recruitment

Screening and consenting will be the same as those described in WP-I. The research team aims to obtain copies of the NICU diaries from all parents whose preterm infants participated in WPI (40 diaries or the number achieved within the recruitment period of 1 year).

##### Inclusion criteria

- Parents of preterm infants recruited in the REPORT-BPD feasibility study
- English speaking

##### Exclusion criteria

- Non-English speaking

#### Data collection and analysis

Once consent is obtained from the parents on the second DAB of their infants, the research team will give the parents a NICU diary along with a study leaflet about ‘how to write a NICU diary’. The diary comprises a small notebook consists of 50 blank pages. A NICU diary writing guide will be given to parents advising what they might write in NICU diary such as reflections, feelings, comments about their child health and progress. There are no limitations on the frequency of notetaking by parents, i.e. they can choose to write as little or as much as possible. On the 10th DAB, the research team will obtain a copy of the parental notes documented in their preterm infants’ NICU diary from the 2nd to 10th DAB, as the original copy is for their keepsake. Therefore, only the records of the NICU diary within the first 10 DABs will be collected. The parental notes written in the NICU diary will be transcribed for analysis.

The transcripts will be imported into the qualitative analysis software programme NVivo to enable the organisation and analysis of textual data. A thematic analysis will be deployed and comprise the following six steps [25]. The first step will involve familiarisation with the text, where two researchers (W Muhsen (WM) and J Latour (JL)) will independently read the transcripts. In the next step, these researchers will independently code the text by allocating text fragments to codes. The codes might be revised during the process of reading the transcripts. In step three, the two researchers will discuss the results of individual codes (sub-themes) and refocus their analysis on identifying potential themes. Step four will involve two levels: level 1 will involve scrutinising the identified themes to generate an accurate thematic map, and in level 2, the researchers will examine whether the generated thematic map accurately reflects the codes extracted from the datasets. The fifth step will involve defining and naming the themes by further refining them. Finally, the sixth step will involve creating the final report, which will be focused on writing a concise, coherent, logical, non-repetitive and interesting account of the data story [25].

### WP-III

This embedded qualitative study, which assesses the experiences of the parents and clinicians about their involvement in the REPORT-BPD study, will provide an important insight into the different aspects of the study, such as the effectiveness of the recruitment process and the acceptability of the procedures [26]. In addition, this qualitative work, as part of the REPORT-BPD study, will be crucial in evaluating the perception of the study participants (parents and clinicians) regarding the study processes and investigations used (e.g. the echo scans), which will be important to inform the design of a larger study [27].

#### Objectives

- To explore the experiences of parents in participating in the REPORT-BPD feasibility study
- To explore the experiences of healthcare professionals in participating in the feasibility study as part of the neonatal research team

#### Study design and settings

This is an additional qualitative research project of the REPORT-BPD study that explores the parents’ and healthcare professionals’ experiences of the REPORT-BPD feasibility study using semi-structured interviews.

This additional qualitative work, as part of the REPORT-BPD study, will be conducted at the regional tertiary NICU, UHP, Plymouth.

#### Recruitment

Purposive stratified sampling of the participants will be undertaken. In qualitative research methods, a sample size of between 5 and 25 participants is generally accepted to reach data saturation depending on the study phenomenon [28]. Therefore, the research team will aim to recruit 10–15 parents and 10–15 healthcare professionals depending on the data saturation. The clinicians’ sample will be made consistent with that of the doctors and nurses involved in the REPORT-BPD study as part of the neonatal research team at any stage from recruitment, enrolment or data collection.

##### Inclusion criteria

Parents’ inclusion criteria

- Their infants are recruited for the REPORT-BPD feasibility study
- They speak English

Healthcare professionals’ inclusion criteria

- A staff member involved in the REPORT-BPD feasibility study procedures, such as screening, recruitment and data collection, as part of the neonatal research team

##### Exclusion criteria

Parents’ exclusion criteria

- Non-English speaking
- Parents whose child has died
- Parents who have withdrawn from the study

Healthcare professionals’ exclusion criteria

- Temporary staff members, such as locum or bank staff

The eligibility assessment of the recruited preterm infants’ parents will be conducted 2–3 days prior to the last echo scan on the 9th day of postnatal life.

The chief investigator (CI) will identify healthcare professionals who participated in the REPORT-BPD feasibility study by reviewing the delegation log register.

Taking the consent of eligible participants to participate in this qualitative research as part of the REPORT-BPD study will not start until 50% of the WP-I and II recruitment target is achieved.

##### Parents’ consent

Eligible parents of preterm infants recruited for the REPORT-BPD feasibility study WP-I and WP-II will be invited by the CI to participate in this qualitative research as part of the REPORT-BPD study. A PIS for this qualitative research project will be provided to the parents during an initial brief face-to-face conversation with the CI. If the parents are interested in participating, the CI will meet them to address their queries and ask them to sign the ICF. Interviews will be conducted within 7 days of obtaining their consent.

##### Clinicians’ consent

Eligible clinicians involved in the initial phases of the REPORT-BPD feasibility study will be invited to participate in this qualitative research by the CI and given the study PIS. The staff members who express their interest in participating in this qualitative research will meet the CI so that their queries can be answered. They will be asked to sign the ICF if they agree to participate. The interviews will be conducted within one month of obtaining their consent.

#### Data collection and analysis

Semi-structured interviews will be conducted to ensure freedom for participants to express their views and to provide an opportunity for the interviewer to ask in-depth questions [29]. The semi-structured interview style will be used due to its exploratory nature with more flexibility compared to a structured interview. It is also more standardised than an unstructured interview [30]. The Cl aims to recruit between 10 and 15 parents and 10 and 15 clinicians depending on the saturation. In the 10th interview, data saturation will be assessed. Data saturation is defined as acquiring enough information to reproduce the study, when the ability to obtain additional information has been accomplished and when further coding is no longer possible [31].

##### Parents’ interviews

Semi-structured interviews will be conducted in a quiet and private place in the NICU at a time convenient for parents.

Both parents will be invited. When both parents are recruited, they will be interviewed separately to obtain individual perspectives and both parents might be unwilling to leave their infant’s bed space simultaneously. The study team will aim to have a balanced recruitment of both parents in the study, since fathers historically are less likely to consent for an interview [32].

##### Clinicians’ interviews

Similar to the parents, the interviews will be semi-structured. The CI will conduct one-to-one interviews in a quiet and private place in the NICU at a time convenient for the recruited clinicians.

Since many team members will be available to look after the study infants during the study procedure, it is important to gather the information from various clinicians, such as nurses, advanced nurse practitioners and doctors at different levels of seniority. The staff members will be identified through the delegation log.

The parents’ and health professionals’ interviews are expected to last approximately 30–45 min using the semi-structured interview guidelines. The interviews will be audio-recorded and transcribed by the CI. Field notes will be completed immediately after the interviews, along with a reflective diary of how the interviewer felt during the interview, which will be used during the data analysis.

Interview guides were developed using the concepts identified in a literature review by O’Cathain et al. [18].

##### Member checking

‘Member checking’ will be used in this phase of the research project to enhance credibility of the qualitative data of WP-III. This method is used to augment the trustworthiness of the extracted data [33].

The focus will be on member checking of the transcripts verbatim only; thus, each participant interview will provide the opportunity to check the transcripts, make amendments, add explanations and see if it is a true reflection of their experiences. Member checking will not be performed on the analysis of the data report for the following reasons. First, the data analysis report will not be available until several months after the data collection (i.e. when the preterm infants are discharged); this might increase the loss to follow-up risk [33]. Second, the process is quite time-consuming for the participants [33]. This might be challenging for the parents who are looking to spend quality time with their children after a lengthy stay at the neonatal unit. Third, sending data analysis reports to the participants might remind them of previous difficult times, which raises ethical concerns as researchers should aim to diminish distress to participants [33].

The member checking process for the interview verbatim transcripts will be performed by giving the participants hard copies of the transcripts to review within 1–2 weeks after their semi-structured interview.

These transcripts will be imported into NVivo to enable the organisation and analysis of textual data. A thematic analysis will be deployed and comprise six steps [25], as detailed in the WP-II data collection and analysis section.

The analysis of interviews of WPIII will enable the study team to unveil any inadequacies of the study processes, such as screening and recruitment, identified by the parents and healthcare professionals so adjustments can be implemented to augment the recruitment and retention rates of the future definitive study.

Study recruitment will be conducted over 18 months. WP-I and WP-II recruitment will be conducted during this period, while WP-III recruitment will be conducted during the second half. The results are expected to be submitted for publication by mid-2024. The outcome measures are detailed in Table 3.

**Table 3:** Outcome measures for all the three WPs

Work package	Outcome measures
**Work package—I**	• Establishing sensitive and feasible echo parameters for detecting right ventricle dysfunction associated with early BPD pulmonary vascular changes • Suitability of eligibility criteria and sample characteristics • Fidelity to the study procedures such as recruitment, data collection including echo scan performance at the set time points • Recruitment, accrual, and retention rates
**Work package—II**	• To gain an understanding of how strong the correlation between the parents’ views of their infant’s health development during the first 10 days of postnatal life and the findings of echo scans at the same period
**Work package—III**	• To gain an understanding of parents and healthcare professionals in participating in the study and identify barriers and enabling factors to inform the methods a full trial

## Discussion

The current scientific literature shows that early BPD starts in the first WAB of the affected preterm infants, with the respiratory function decline manifesting at the 7th day of postnatal life [17]. In addition, animal research findings support the vascular theory, indicating that the vascular changes in early BPD start before the pulmonary parenchymal ones [4]. These findings form the foundation for structuring the proposed study. The research team will perform two echo scans two days before and after the 7th DAB. This will allow a closer examination of RV functional changes in preterm infants with early BPD compared to healthy ones.

The strengths of this study can be summarised as follows. First, it uses two echo scans around the timepoint of change in the pulmonary function. Second, it uses only non-invasive intervention (echo scans). Our tertiary NICU is well-positioned to conduct the study since our experienced neonatal team regularly performs haemodynamic assessments with functional neonatal echo scans. Third, a combination of advanced technologies will be used to assess the RV function (e.g. TDI and speckle tracking). The proposed study will not only highlight the most sensitive echo parameters through the study analysis but also help in identifying if one echo scan, either day 5 or 7 of postnatal life, is adequate to diagnose the early BPD-associated RV dysfunction.

The functional echocardiographic examinations of ductus arteriosus and pulmonary arterial pressure will allow the study team to investigate its effect on the echo parameters related to the RV function in preterm infants with BPD and controls.

The main limitation of the study is that it is an observational study since a randomised controlled trial is not an appropriate design to answer our research question.

To the best of our knowledge, this is the first study performing two echo assessments at 5th and 9th DABs using a combination of the most recent cardiac technologies to analyse the acquired neonatal echo scans. The study findings will allow the formulation of an early BPD predictive model. The future plan is to test this model in a large multi-centre trial.

## Data Availability

Not applicable.

## Abbreviations

BPD: Broncho-pulmonary dysplasia
CI: Chief investigator
DAB(s): Day(s) after birth
Echo: Echocardiogram
GCP: Good clinical practise
ICF: Informed consent form
NICU: Neonatal intensive care unit
NNAP: National Neonatal Audit Programme
PDA: Patent ductus arteriosus
PFO: Patent foramen ovale
PIS: Participant information sheet
PMA: Post-menstrual age
RV: Right ventricle
UHP: University Hospital Plymouth NHS Trust
WP: Work package
WAB(s): Week(s) after birth
